# Identification of AICD-associated transcriptomic markers in major depressive disorder

**DOI:** 10.3389/fpsyt.2026.1782515

**Published:** 2026-07-03

**Authors:** Shengjie Xiong, Lixin Liao, Meng Chen, Rengde Peng, Quan Luo, Qing Gan, Weiping Yang

**Affiliations:** 1 Department of Psychiatry, West China School of Medicine, Sichuan University, Sichuan University Affiliated Chengdu Second People’s Hospital, Chengdu Second People’s Hospital, Chengdu, Sichuan, China; 2 Department of Obstetrics and Gynecology, Chengdu Second People’s Hospital, Chengdu, Sichuan, China; 3 Department of Urology, Chengdu Second People’s Hospital, Chengdu, Sichuan, China; 4 Department of Traditional Chinese Medicine (TCM), Chengdu Second People’s Hospital, Chengdu, Sichuan, China; 5 Department of Emergency, Chengdu Second People’s Hospital, Chengdu, Sichuan, China; 6 Fuqing Road Community Health Center, Chenghua District, Chengdu, Sichuan, China

**Keywords:** AICD-associated markers, immune infiltration, machine learning, major depressive disorder, transcriptomic signatures

## Abstract

**Background:**

Major Depressive Disorder (MDD) is a complex mental disorder with unclear molecular mechanisms. This study aimed to identify key genes associated with ATP-induced cell death (AICD) in MDD and elucidate their roles in disease pathogenesis.

**Methods:**

MDD transcriptome data were obtained from public databases. Genes associated with AICD-related pathways were identified through differential expression analysis, weighted gene co-expression network analysis (WGCNA), and machine learning algorithms. A diagnostic nomogram was constructed and validated. Functional enrichment, immune infiltration, regulatory network, and molecular docking analyses were performed to explore biological functions and therapeutic potential. Gene expression was validated using RT-qPCR.

**Results:**

MRPL53 and RPL24 were identified as key genes with excellent diagnostic performance (AUC > 0.7 in both cohorts). The nomogram based on these genes demonstrated high accuracy (AUC = 0.967 in training cohort). Gene set enrichment analysis revealed significant enrichment in ribosome, neuroactive ligand-receptor interaction, and oxidative phosphorylation pathways. Immune infiltration analysis showed substantial alterations in the MDD immune microenvironment, with key genes strongly correlating with specific immune cell populations. Molecular docking preliminarily suggested hydralazine as a candidate compound with in silico binding affinity to MRPL53 (-5.0 kcal/mol) and RPL24 (-5.7 kcal/mol), warranting further experimental validation in cellular and animal models. RT-qPCR validation confirmed the bioinformatics findings in clinical samples (p < 0.05).

**Conclusion:**

MRPL53 and RPL24 were identified as candidate AICD-associated transcriptomic markers in MDD, providing novel insights into MDD pathogenesis and suggesting hydralazine as a potential therapeutic candidate. However, the training cohort (whole blood) and validation cohort (PBMCs) differ in sample type, which may affect cross-cohort comparability and should be considered when interpreting the reproducibility of these findings.

## 1 Introduction

Major depressive disorder (MDD) is one of the most common mental illnesses, affecting over 350 million people globally and characterized by persistent sadness, anhedonia, and, in severe cases, suicidal ideation (1). The primary clinical manifestations include persistent sadness, loss of pleasure and interest, feelings of hopelessness and worthlessness, high recurrence rates, and potentially suicidal ideation (2, 3). Despite advances in pharmacological therapies, 30% to 50% of patients exhibit treatment resistance, and the underlying mechanism has not yet been fully elucidated (4, 5).

Although the pathogenesis of MDD is not yet fully understood, it is widely believed that MDD is associated with multiple pathogenic factors including genetic predispositions, social stressors, neurotransmitters, hormones, oxidative stress, and various cytokines (6). Specifically, depressive behaviors are closely related to purine signaling, where the activation of P2X7 purine receptors (P2X7R) in glial cells can exacerbate depressive-like symptoms (7). Studies show that chronic stress-induced ATP dysregulation affects P2X7R-mediated neuronal activity, and chronic sleep deprivation can increase extracellular ATP levels, stimulate P2X7R, and downregulate 5-HT2BR expression, thereby causing depression-like behaviors (8). P2X7R plays a crucial role in ATP-induced cell death (AICD). Upon binding to P2X7R, extracellular ATP induces calcium influx, which triggers mitochondrial dysfunction, ROS production, NLRP3 inflammasome activation, and ultimately cell death (9). ATP activates the P2X7 receptor (P2X7R), opening ion channels and promoting Ca²^+^ influx, which leads to mitochondrial dysfunction, ROS production, and activation of the NLRP3 inflammasome, ultimately resulting in cell death (9, 10).

Clinical studies have found that adolescents with MDD have elevated pro-inflammatory cytokines and NLRP3 levels in their serum, which significantly decrease after drug treatment (11). Inflammasome activation leads to Caspase-1 maturation and subsequent IL-1β and IL-18 release. Caspase-1-deficient mice exhibit reduced depression and anxiety-like behaviors, and Caspase-1 absence prevents chronic stress-induced depressive behaviors (12). Animal models also demonstrate that increased ATP release is associated with depressive-like behaviors (13). However, while current research predominantly focuses on neuronal apoptosis, the specific role and mechanisms of ATP-dependent cell death in MDD pathogenesis remain unclear.

This study utilized MDD-related transcriptome datasets to investigate the underlying molecular mechanisms. Through gene differential expression analysis, weighted gene co-expression network analysis (WGCNA), and machine learning, key genes potentially associated with AICD-related pathways in MDD were identified. The functional characteristics, immune features, and molecular regulatory networks of these key genes were investigated, suggesting their involvement in mitochondrial and ribosomal dysregulation rather than direct AICD axis components.

## 2 Materials and methods

### 2.1 Data source

The training cohort was sourced from the Gene Expression Omnibus (GEO) database (https://www.ncbi.nlm.nih.gov/geo/) under accession number GSE52790 (GPL17976 platform), comprising peripheral blood samples from 12 major depressive disorder (MDD) patients and 10 normal controls. The validation cohort GSE38206 (GPL13607 platform), containing 9 MDD patients and 9 control peripheral blood mononuclear cell samples, was downloaded from the GEO database. Of note, the training cohort used whole blood while the validation cohort used PBMCs, a difference in sample type that introduces biological heterogeneity in cellular composition and may confound cross-cohort comparisons, particularly for immune infiltration analyses. The 37 genes pertinent to ATP-induced cell death (AICD) were derived from reference (14), and the full list is provided in **Supplementary Table 1**. Expression matrices and platform annotation files were downloaded from the GEO database. Expression normalization was performed automatically using log2(expr + 1) if values exceeded 100, showed excessive range, or abnormal distribution. Probe IDs were converted to gene symbols according to the corresponding GPL platform annotation. For multiple probes mapping to the same gene, the probe with the highest mean expression was retained. Only protein-coding genes were included for subsequent analysis. Sample information was extracted and cleaned; samples without group information were excluded, and samples were reordered by group for downstream analysis.

### 2.2 Differential expression analysis

Differentially expressed genes (DEGs) between MDD and control groups were identified using the “limma” package (v 3.52.4) (15). Genes with |log2FC| > 0.25 and adjusted p < 0.05 were considered significant. Volcano and heat maps were created using the “ggplot2” (v 3.4.1) (16) and “ComplexHeatmap” (v 2.14.0) (17) packages.

### 2.3 AICD-related gene scoring and statistical analysis

AICD-related gene scores were computed using the “GSVA” package (v 1.46.0) (18). The Wilcoxon rank-sum test was used to compare score differences between MDD and control groups (p < 0.05), with results visualized using boxplots.

### 2.4 Weighted gene co-expression network analysis

WGCNA was performed using the “WGCNA” package (v 1.71) (19) to identify gene modules associated with AICD phenotypes. Hierarchical clustering based on Euclidean distance was conducted for all samples. The soft threshold (power) was selected to achieve scale-free topology with R² ≥ 0.8. The minimum gene count per module was set at 50, with mergeCutHeight at 0.25. Module-trait association analysis correlated module genes with AICD scores, selecting modules with a correlation coefficient > 0.3 and p < 0.05.

### 2.5 Acquisition of candidate genes

Subsequently, to pinpoint candidate genes linked to AICD, the “ggvenn” package (v 0.1.9) (20) was utilized to create visualizations and extract overlapping genes between the DEGs and module genes. These intersecting genes were then marked as candidate genes for further functional validation and mechanistic investigation.

### 2.6 Functional enrichment analysis

Gene Ontology (GO) and Kyoto Encyclopedia of Genes and Genomes (KEGG) enrichment analyses were performed using the “clusterProfiler” package (v 4.7.1.003) (21) with p < 0.05 as the significance threshold. GO analysis covered biological process (BP), molecular function (MF), and cellular component (CC) categories. The top 5 enriched terms from each category were visualized.

### 2.7 Protein-protein interaction network

PPI networks of candidate genes were constructed using the Search Tool for the Retrieval of Interacting Genes (STRING) database (https://string-db.org/). A confidence level of 0.4 was chosen as the screening threshold. The network data obtained were then saved in tab-separated values (TSV) format and subsequently loaded into the Cytoscape software (version 3.7.2) (22). for topological analysis and visualization. To refine candidate feature genes from the PPI network, molecular complex detection was performed using the MCODE plugin within Cytoscape software. The parameters for clustering were established with the following settings: a degree cutoff of 2, a node score cutoff of 0.2, a k-core value of 2, and a maximum depth of 100, ensuring identification of densely interconnected subnetworks while maintaining biological interpretability. The top-ranked module generated by the MCODE plugin was selected based on its clustering score and topological significance. Genes within this module were extracted as candidate feature genes for further analysis.

### 2.8 Machine learning

LASSO analysis was implemented using the “glmnet” package (v 4.1.4) (23) with five-fold cross-validation to determine the optimal regularization parameter (λ) based on lambda. min. Random forest modeling used the “randomForest” package (v 4.7.1.1) (24). with 100 bootstrap iterations. The optimal number of trees (ntree) was determined by monitoring out-of-bag (OOB) error rates. The top 10 genes ranked by Gini coefficient importance were selected as feature genes. Genes identified by both LASSO and random forest were retained as final feature genes using the “ggvenn” package.

### 2.9 Evaluation of diagnostic potential for feature genes

The diagnostic performance of feature genes in differentiating MDD samples from normal controls was systematically assessed using receiver operating characteristic (ROC) curve analysis. Specifically, the “pROC” package (v 1.18.0) was utilized to calculate the area under the curve (AUC) for both the training cohort and the validation cohort. For gene selection criteria, candidate key genes were required to simultaneously satisfy two conditions: 1) AUC > 0.7 in both training and validation cohort, and 2) AUC ≠ 1 in either dataset to exclude perfect classifiers potentially caused by data artifacts.

### 2.10 Expression analysis of candidate key genes

Comprehensive analyses of the distinct expression profiles of candidate key genes were performed between the MDD and control groups in both the training and validation cohorts, utilizing the Wilcoxon rank-sum test with a significance threshold of p < 0.05. Genes exhibiting significant expression discrepancies between MDD and control specimens (p < 0.05) and uniform expression profiles across the two cohorts were designated as key genes.

### 2.11 Nomogram construction for risk prediction

A nomogram was constructed using the “rms” package (v 6.5.0) (25). to visualize the collective predictive influence of key genes on MDD susceptibility. ROC analysis was performed using the “pROC” package to assess discriminatory power, with AUC > 0.7 indicating moderate diagnostic efficacy and AUC > 0.9 denoting high precision. Calibration curves were generated using the “ResourceSelection” package (v 0.3.5) (26) combined with Harrell’s optimism-corrected bootstrap method to assess concordance between predicted probabilities and actual outcomes; 1000 bootstrap resamplings were implemented to perform bias correction and quantify the predictive error of the nomogram model, with MAE < 0.1 signifying high accuracy. Decision curve analysis (DCA) was conducted using the “rmda” package (v 1.6) (27) to evaluate net clinical benefit.

### 2.12 Gene set enrichment analysis and gene set variation analysis

GSEA was performed using the “c2.cp.kegg.v2023.1.Hs.symbols” gene set from MSigDB (https://www.gsea-msigdb.org/gsea/msigdb). Spearman correlation coefficients were calculated for each key gene using the “psych” package (v 2.1.6) (https://CRAN.R-project.org/package=psych) (28). Genes were ranked by correlation coefficients, and GSEA was conducted using “clusterProfiler” with thresholds of |NES| > 1, p < 0.05, and q-value < 0.25. The top 10 enriched pathways were visualized using the “enrichplot” package (v 1.18.3) (29).

GSVA assessed pathway activity differences between MDD and control groups. ssGSEA scores were computed using the “GSVA” package, and differential pathway activity was evaluated using “limma” with thresholds of p < 0.05 and |t| > 2. Results were visualized as heatmaps using “ggplot2”.

### 2.13 Immune infiltration analysis

Utilizing the ssGSEA algorithm from the “GSVA” package, immune cell scores for each sample were computed using the training cohort. The Wilcoxon rank-sum test was then applied to evaluate the differences in immune cell abundance between the MDD and control groups (p < 0.05), identifying the immune cells that varied. The “psych” package was systematically applied to explore the associations between the differential immune cells and their connections with key genes (|cor| > 0.3, p < 0.05).

### 2.14 Construction of molecular regulatory interaction networks

RNA-binding proteins (RBPs) interacting with key genes were predicted using the StarBase v3.0 database (https://rnasysu.com/encori/). Transcription factors (TFs) were identified via the JASPAR database (https://jaspar.genereg.net/) embedded within NetworkAnalyst (https://www.networkanalyst.ca/). Interaction data were visualized using Cytoscape. Functional similarity networks were constructed using the GeneMANIA platform (https://genemania.org/), incorporating co-expression patterns, physical interactions, and shared protein domains from 11 databases.

### 2.15 Chromosomal localization analysis and protein structure prediction

Chromosomal localization of key genes was performed using the “RCircos” package (v 1.2.2) (30). Three-dimensional protein structures were predicted using AlphaFoldDB (https://alphafold.com/download), with canonical amino acid sequences obtained from UniProt (https://www.uniprot.org/).

### 2.16 Drug target prediction and molecular docking validation

Potential drugs targeting key genes were predicted using the Drug Signatures Database (DSigDB, https://dsigdb.tanlab.org/DSigDBv1.0/) via the “enrichR” package (v 3.2) (31). Irrelevant or biologically implausible compounds were excluded prior to network construction. Drug-gene interaction networks were visualized using Cytoscape. Molecular docking was performed to evaluate binding affinities between key genes and predicted drugs. Crystal structures of key genes were obtained from UniProt (https://www.uniprot.org/), while drug structures were downloaded in SDF format from PubChem (https://pubchem.ncbi.nlm.nih.gov/). Docking analysis was conducted using Cad Lab (https://cadd.labshare.cn/cb-dock2/php/index.php). All databases were accessed on 24–25 March 2025.

### 2.17 Reverse transcription-quantitative polymerase chain reaction

Samples of MDD patients and healthy controls were collected at Chengdu Second People’s Hospital. The study received ethical approval from The Medical Ethics Review Committee of Chengdu Second People’s Hospital. Total RNA was extracted using TRIzol reagent (Vazyme, R401-01, China) following the manufacturer’s instructions (n = 5 per group). RNA concentration and purity were measured using NanoPhotometer N50 (Implen). Reverse transcription was performed using Hifair^®^III 1st Strand cDNA Synthesis SuperMix Kit (Yeasen Biotechnology, 11141ES60, China). The cDNA products were diluted 5–20 times with DNase/RNase-free water before qPCR. qPCR reactions were carried out using 2×Universal Blue SYBR Green Master Mix (Servicebio, G3326-05, China) on a CFX Connect Real-Time PCR Detection System (BIO-RAD, USA). Primer sequences are detailed in **Supplementary Table 2**. All reactions were performed in technical triplicate. Relative gene expression levels were determined using the 2^-ΔΔCt method with GAPDH as the internal control. Statistical analyses were performed using GraphPad Prism software (v 8.0) (32).

### 2.18 Statistical analysis

Bioinformatics analyses were conducted using R (v 4.2.3). Comparative analysis of data from different groups was carried out using the Wilcoxon test, and the difference between different groups in the RT-qPCR was determined using the t-test. A p-value less than 0.05 was deemed statistically significant.

## 3 Results

### 3.1 Acquisition of 696 DEGs for MDD

**Figure 1:**
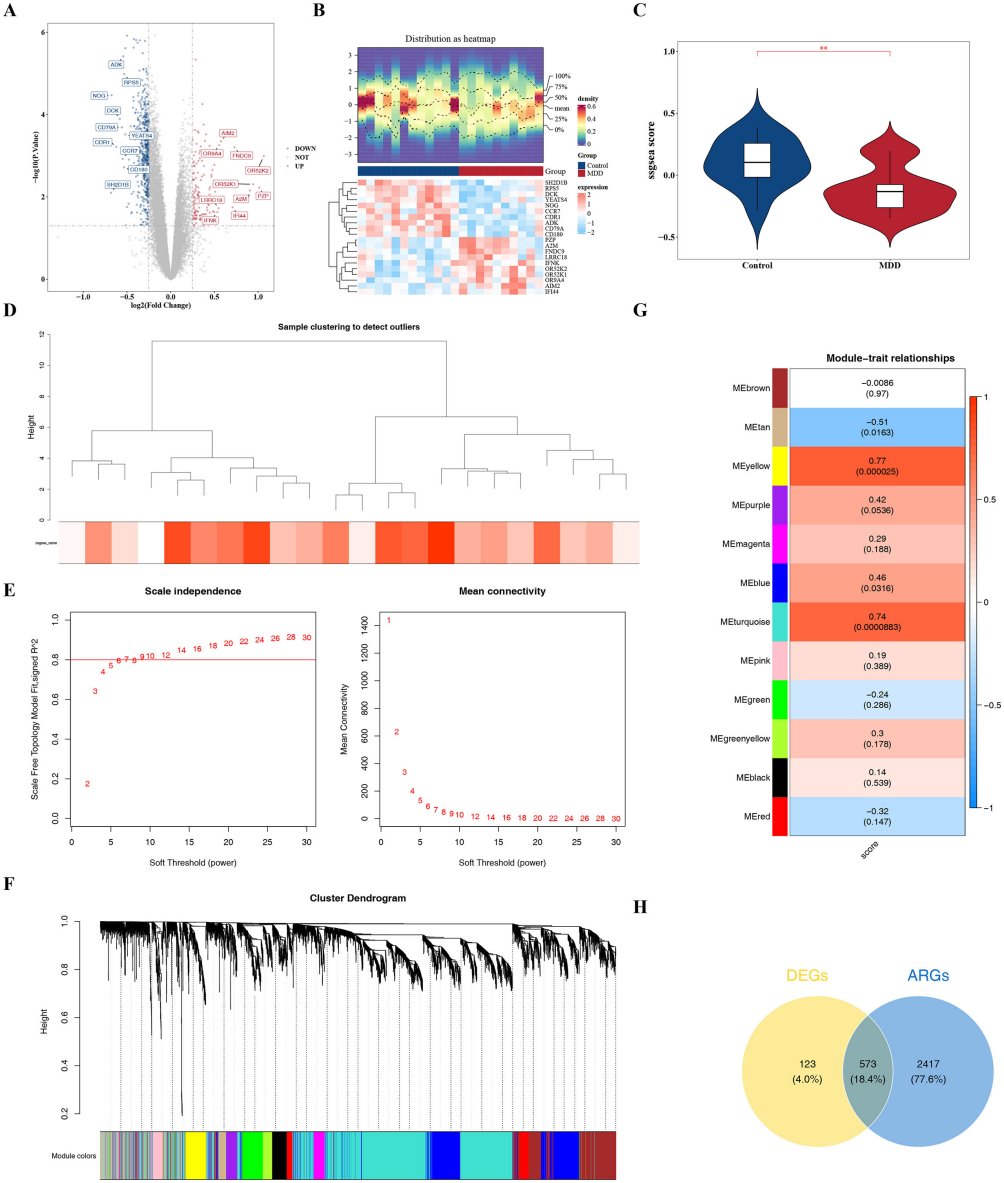
Identification of differentially expressed genes and WGCNA analysis in MDD. **(A)** Volcano plot of 696 DEGs (|log_2_FC| > 0.25, p < 0.05). Red: upregulated; blue: downregulated. **(B)** Heatmap of DEG expression patterns. **(C)** Violin plot comparing AICD-related gene scores between control and MDD groups (**p < 0.01). **(D)** Sample clustering dendrogram. **(E)** Soft-thresholding power analysis (power = 9, R² = 0.8). **(F)** Gene dendrogram and module assignment. **(G)** Module-trait relationship heatmap. **(H)** Venn diagram showing 573 candidate genes.

To explore transcriptional changes linked to MDD, a differential expression analysis was executed comparing MDD patients with healthy controls in the training cohort. A total of 696 DEGs were pinpointed with a threshold of |log_2_FC| > 0.25 and p < 0.05, where 129 genes were found to be upregulated and 567 genes downregulated in MDD samples in contrast to control samples (**Figures 1A, B**).

### 3.2 Identifying the key co-expression modules linked to AICD in MDD

MDD samples showed significantly lower AICD-related gene scores compared to controls (p < 0.01) (**Figure 1C**). WGCNA was conducted to identify modules associated with the AICD phenotype. Sample clustering revealed no outliers (**Figure 1D**), and all samples were retained for analysis. A soft threshold of 9 was selected to achieve a scale-free topology fit index (R² = 0.8) with optimal average connectivity (**Figure 1E**). This yielded 12 co-expression modules after merging similar modules and excluding unassigned genes in the gray module (**Figure 1F**). Correlation analysis identified 4 significant modules (|cor| > 0.3, p < 0.05): the yellow module (232 genes, cor = 0.77), blue module (800 genes, cor = 0.46), and turquoise module (1,891 genes, cor = 0.74) showed positive correlations, while the brown module (67 genes, cor = -0.51) displayed negative correlation (**Figure 1G**). These modules comprised 2,990 genes designated as module genes. The intersection of module genes and DEGs yielded 573 candidate genes (**Figure 1H**).

### 3.3 Exploration of the functions and pathways connected with the candidate genes

**Figure 2:**
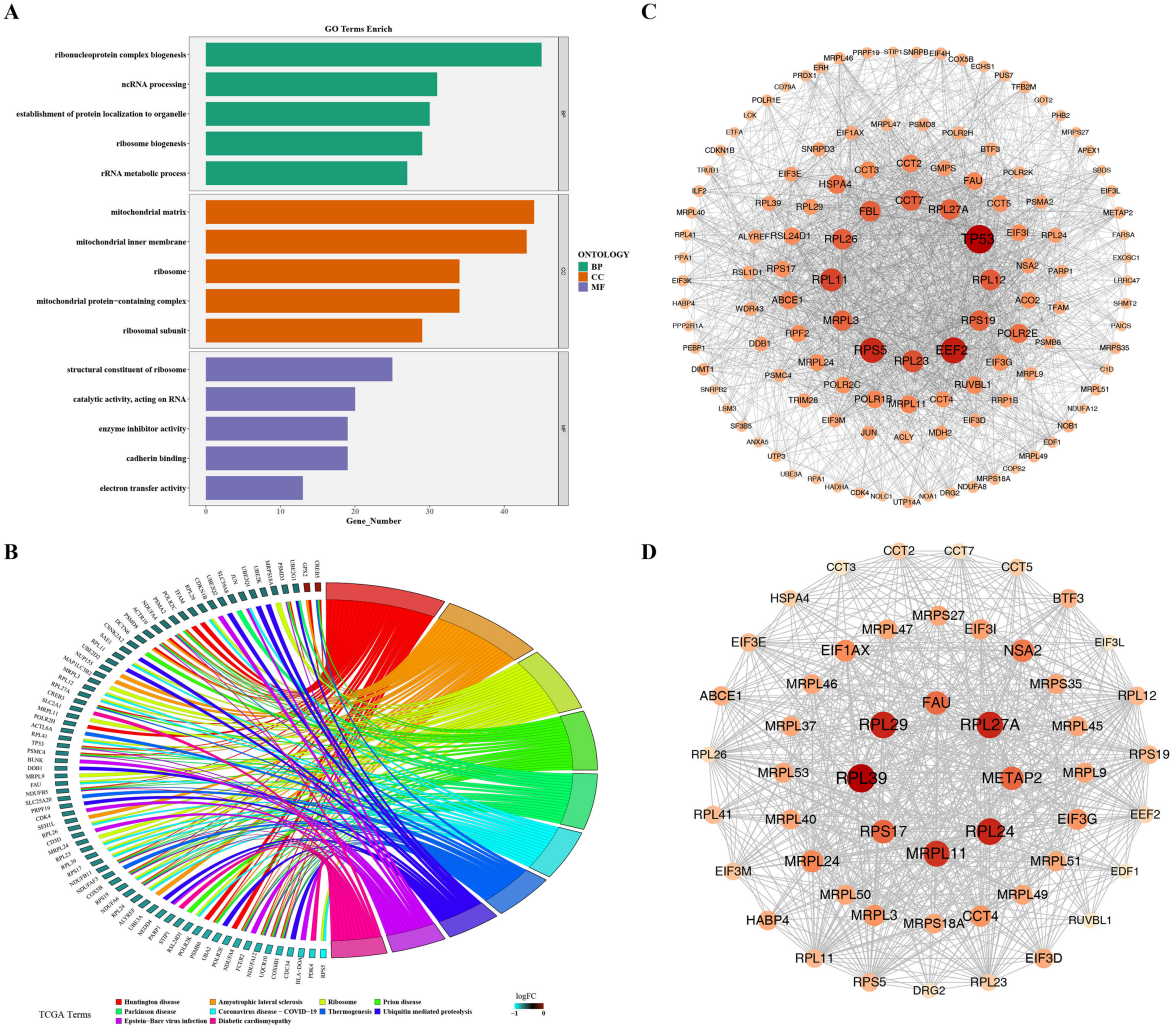
Functional enrichment and protein-protein interaction network analysis of candidate genes. **(A)** GO enrichment analysis showing top 5 terms in BP, CC, and MF categories (p < 0.05). **(B)** KEGG pathway enrichment analysis of top 10 pathways (p < 0.05). **(C)** PPI network displaying top 20 hub genes ranked by degree centrality. **(D)** The most significant MCODE module contain 51 candidate feature genes.

GO enrichment analysis revealed significant terms (p < 0.05) across BP, CC, and MF. The top 5 enriched BP terms included ribonucleoprotein complex biogenesis, ncRNA processing, and establishment of protein localization to organelles. For CC, enrichment was observed in the mitochondrial matrix, mitochondrial inner membrane, and ribosome. MF enrichment included structural constituents of the ribosome, catalytic activities involving RNA, and enzyme inhibitor activity (**Figure 2A**). KEGG analysis identified significantly enriched pathways (p < 0.05), with the top 10 including Huntington’s disease, amyotrophic lateral sclerosis, ribosome, prion disease, and Parkinson’s disease (**Figure 2B**). The PPI network comprised 122 candidate genes with 1,597 interaction pairs, with TP53, RPS5, and EEF2 showing the most interactions. The top 20 hub genes ranked by degree centrality were visualized (**Figure 2C**). MCODE identified the first module containing 51 candidate feature genes, including RPL29, RPL39, and MRPL11 (**Figure 2D**).

### 3.4 Identification of feature genes via machine learning algorithms

**Figure 3:**
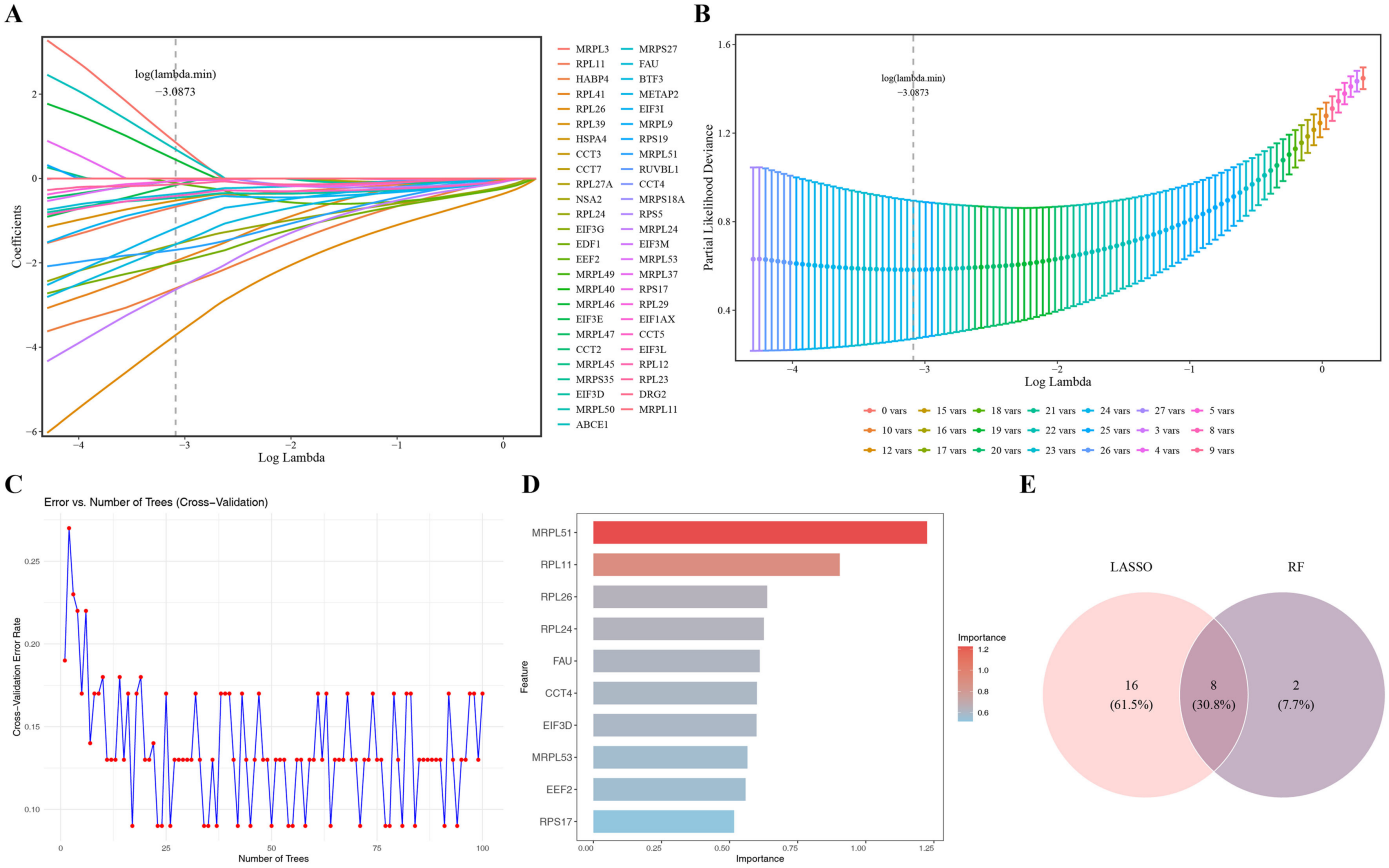
Machine learning-based feature gene selection. **(A)** LASSO regression with 5-fold cross-validation (log[λ.min] = -3.0873). **(B)** LASSO coefficient paths. **(C)** OOB error rate versus number of trees (optimal ntree = 17). **(D)** Variable importance ranking showing top 10 genes by Gini coefficient. **(E)** Venn diagram of 8 overlapping feature genes identified by LASSO and random forest.

Utilizing LASSO analysis with 5-fold cross-validation, 24 genes were identified from the 51 candidate feature genes (MRPL3, RPL11, HABP4, RPL41, RPL26, RPL39, RPL24, EDF1, EEF2, MRPL46, EIF3E, MRPL47, EIF3D, ABCE1, FAU, BTF3, METAP2, EIF3I, MRPL51, MRPL24, MRPL53, RPL29, EIF3L, and RPL23) under the minimum lambda value (log [λ.min] = -3.0873), demonstrating minimal cross-validation error (**Figures 3A, B**). Concurrently, when the value of ntree was 17, the bag error of the model was the smallest. Based on the sorting results, MRPL51, RPL11, RPL26, RPL24, FAU, CCT4, EIF3D, EEF2, RPS17 and MRPL53 were retained (**Figures 3C, D**). The two machine learning algorithms shared 8 feature genes (RPL11, RPL26, RPL24, EEF2, EIF3D, FAU, MRPL51, and MRPL53) (**Figure 3E**).

### 3.5 Identification of key genes

**Figure 4:**
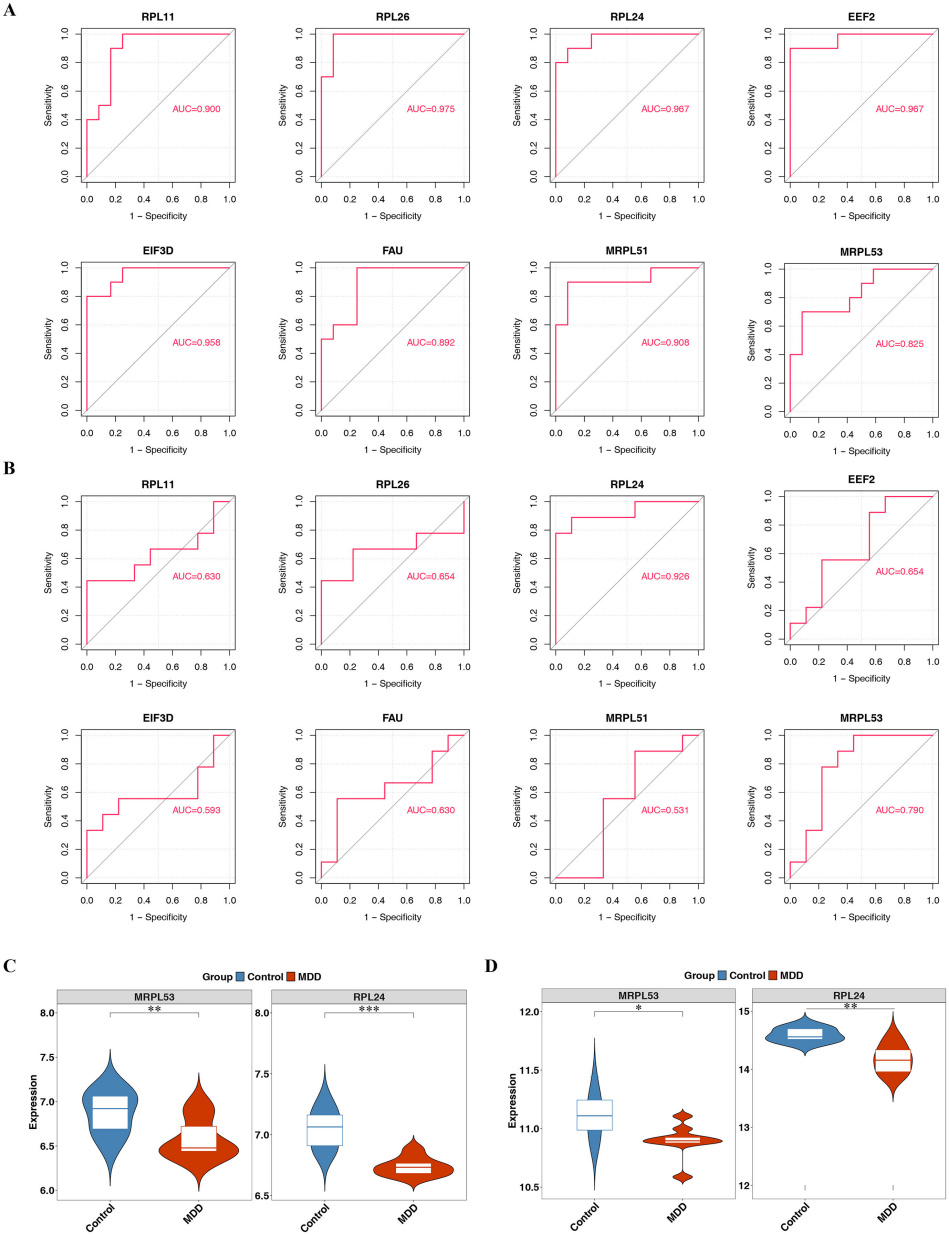
Validation of key genes in training and validation cohorts. **(A)** ROC curves of 8 feature genes in the training cohort (all AUC > 0.7). **(B)** ROC curves of 8 feature genes in the validation cohort. **(C)** Expression levels of MRPL53 and RPL24 in the training cohort showing significant downregulation in MDD (**p < 0.01, ***p < 0.001). **(D)** Expression levels of MRPL53 and RPL24 in the validation cohort confirming consistent downregulation in MDD (*p < 0.05, **p < 0.01).

ROC analysis was performed to assess the diagnostic potential of the 8 feature genes in both training and validation cohorts. In the training cohort, all 8 feature genes demonstrated strong discriminative efficacy with AUC values exceeding 0.7 (**Figure 4A**). However, only MRPL53 (AUC = 0.81) and RPL24 (AUC = 0.78) maintained similar predictive performance (AUC > 0.7) in the validation cohort (**Figure 4B**). These two genes were designated as key genes, meeting the criteria of cross-cohort diagnostic consistency (AUC > 0.7 in both cohorts) and biological plausibility (AUC ≠ 1), though this finding should be interpreted with caution given the sample type differences between whole blood (training) and PBMCs (validation). Differential expression analysis using the Wilcoxon rank-sum test revealed significant downregulation of MRPL53 and RPL24 in MDD samples compared to controls (p < 0.05) (**Figures 4C, D**), with concordant expression patterns across both cohorts.

### 3.6 Creation and assessment of a nomogram to predict the risk of MDD

**Figure 5:**
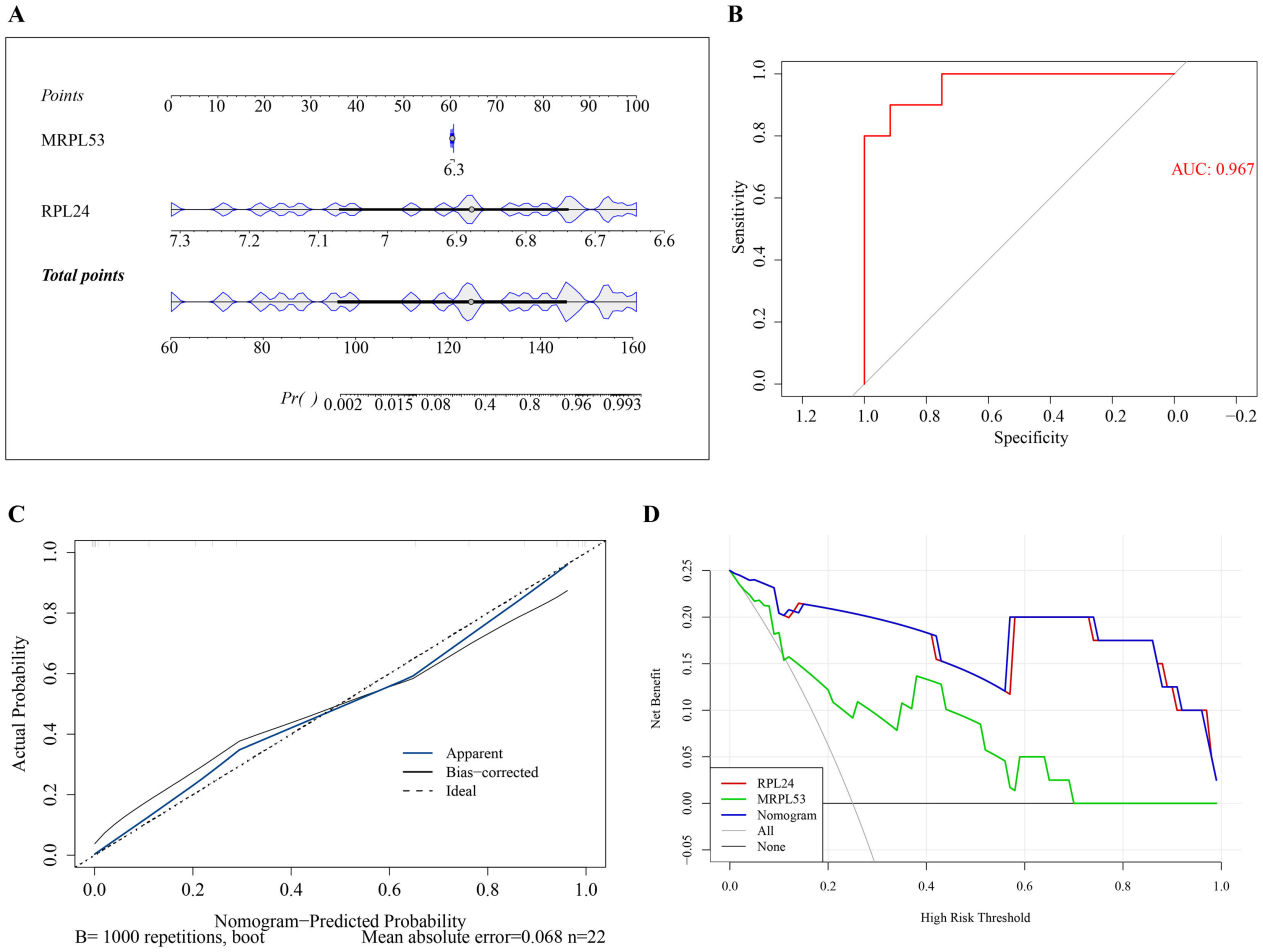
Construction and evaluation of nomogram for MDD risk prediction. **(A)** Nomogram based on MRPL53 and RPL24 expression for predicting MDD risk. **(B)** ROC curve of the nomogram model (AUC = 0.967). **(C)** Calibration curve showing agreement between predicted and observed probabilities (MAE = 0.068). **(D)** Decision curve analysis demonstrating clinical net benefit.

To further assess the clinical significance of the identified key genes (MRPL53 and RPL24), a nomogram model was developed to forecast MDD risk using their expression profiles from the training cohort (**Figure 5A**). The nomogram suggested that the incidence of MDD rises as the total score increases. ROC analysis demonstrated strong discriminatory power, with an AUC of 0.967 in the training cohort (**Figure 5B**). Calibration curve analysis revealed a slight deviation between predicted and observed MDD probabilities (mean absolute error (MAE) = 0.06), indicating high model precision. (**Figure 5C**). To minimize overfitting, internal validation was conducted using Harrell’s optimism-corrected bootstrap with 1000 resamplings, producing a bias-corrected MAE of 0.072. Both MAE values were below a predefined acceptable threshold of 0.1, suggesting acceptable calibration and internal stability within this cohort (**Supplementary Figure 1**). DCA further validated the clinical utility of the nomogram, exhibiting significant net benefit over a broad spectrum of threshold probabilities (**Figure 5D**).

### 3.7 Functional enrichment of key genes in MDD pathogenesis

**Figure 6:**
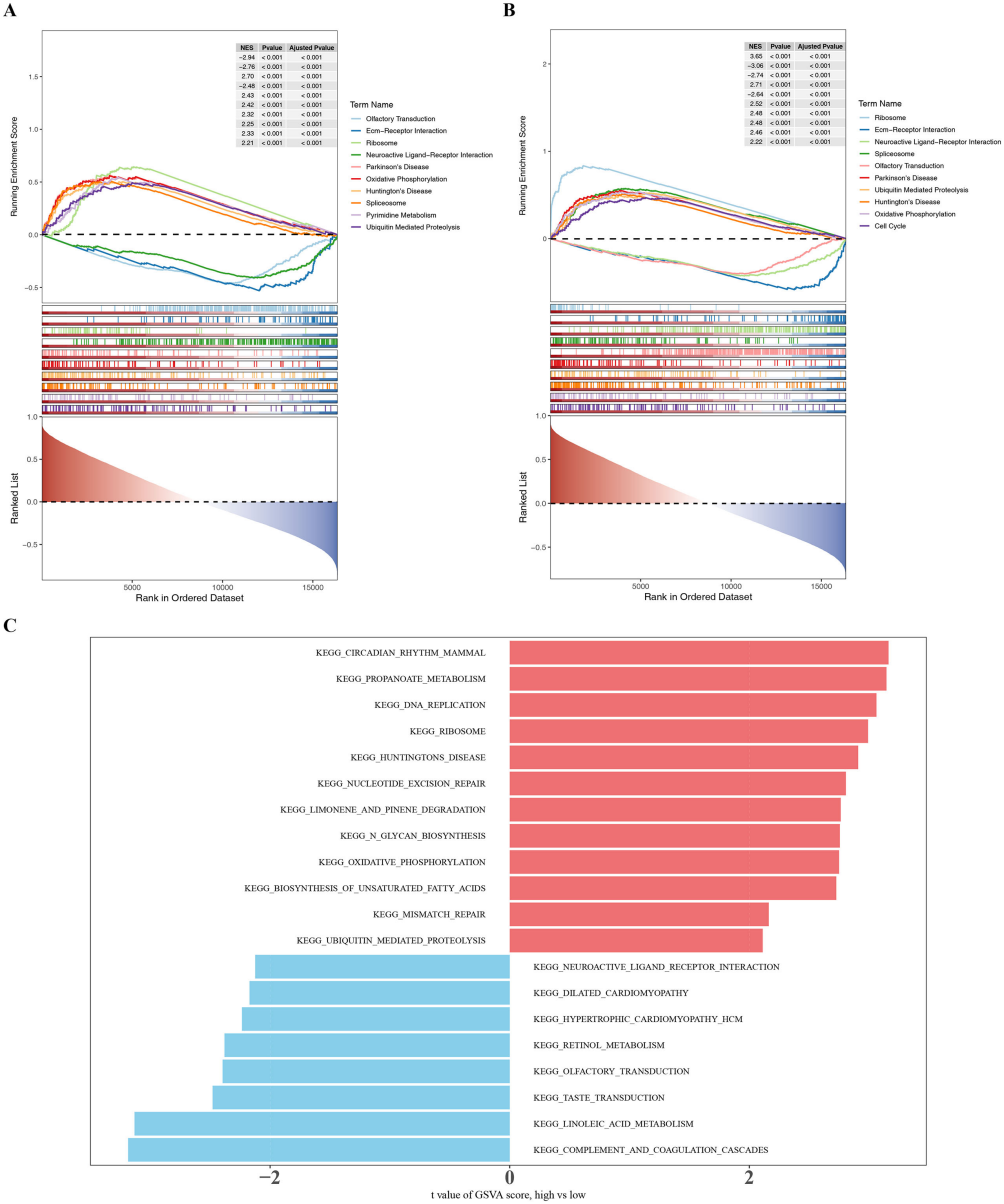
Gene set enrichment analysis and pathway dysregulation in MDD. **(A, B)** GSEA showing top 10 significantly enriched KEGG pathways for MRPL53 **(A)** and RPL24 **(B)** (|NES| > 1, p < 0.05, q < 0.25). **(C)** Heatmap of differential pathway activity between MDD and control groups based on GSVA (p < 0.05, |t| > 2). Red: activated pathways; blue: suppressed pathways.

Within GSEA, the results showed MRPL53 and RPL24 were significantly enriched in ECM-receptor interaction, ribosome, neuroactive ligand-receptor interaction, spliceosome, olfactory transduction, Parkinson’s disease, ubiquitin mediated proteolysis, Huntington’s disease, and oxidative phosphorylation (**Figures 6A, B**). This suggests that key genes could be involved in the onset and progression of MDD by engaging in pathways that were closely linked to the disease’s pathology. Further, this might provide a basis for targeted regulation of these key pathways, thereby enabling potential targeted interventions for MDD.

### 3.8 Pathway dysregulation in MDD

Compared to controls, 39 pathways demonstrated significant differential activity in MDD (p < 0.05, |t| > 2), comprising 31 activated and 8 suppressed pathways (**Figure 6C**). Activated pathways included citrate cycle/TCA cycle (t = 4.32), phospholipid metabolism (t = 3.89), and DNA replication (t = 3.65), suggesting enhanced energy production and cellular proliferation. Suppressed pathways included complement and coagulation cascades (t = -3.21), linoleic acid metabolism (t = -2.98), and taste transduction (t = -2.76), indicating impaired immune regulation and sensory signaling. The TCA cycle hyperactivity aligns with mitochondrial dysfunction in mood disorders (33), while suppressed complement pathways reflect dysregulated neuroinflammatory responses. The suppression of taste transduction pathways corroborates clinical observations of sensory perception deficits in MDD patients (34). These pathway-level perturbations highlight multifactorial MDD pathophysiology involving metabolic reprogramming, immune dysregulation, and neuro-sensory impairment.

### 3.9 Immune cell infiltration dynamics in MDD pathogenesis

**Figure 7:**
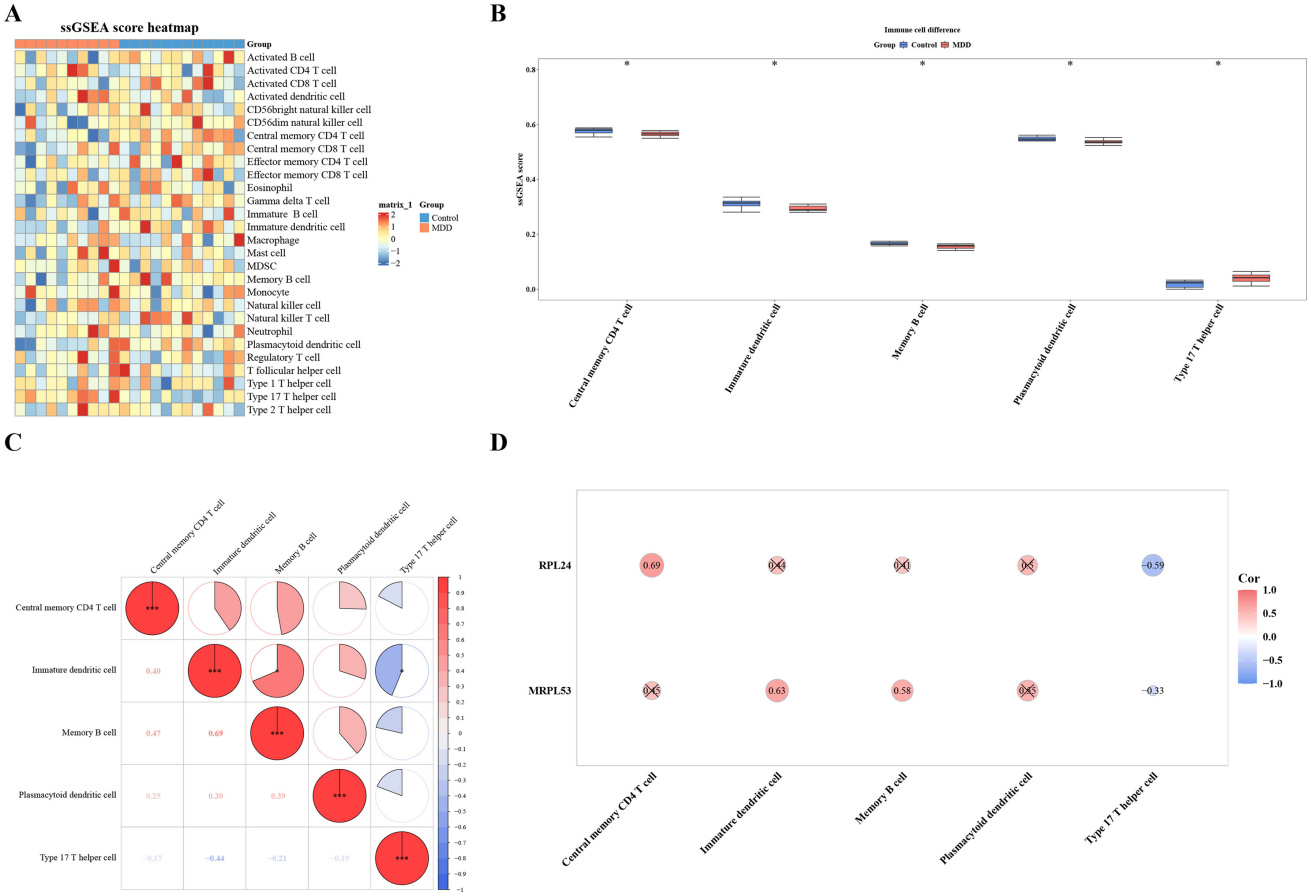
Immune infiltration analysis in MDD. **(A)** Heatmap of ssGSEA scores for 28 immune cell types across MDD and control samples. **(B)** Boxplots showing five significantly differential immune cell populations between MDD and controls (Wilcoxon test, *p < 0.05). **(C)** Correlation matrix among the five differential immune cell types. **(D)** Correlation heatmap showing associations between key genes (MRPL53, RPL24) and differential immune cells. Circle size and color intensity represent correlation strength; X indicates non-significant correlations (p ≥ 0.05).

ssGSEA-based immune infiltration analysis identified 5 significantly different immune cell types between MDD and control samples (p < 0.05): central memory CD4 T cells, immature dendritic cells, memory B cells, plasmacytoid dendritic cells, and type 17 T helper cells (**Figures 7A, B**). Intercellular correlation analysis revealed a positive correlation between memory B cells and immature dendritic cells (cor = 0.69, p < 0.05), while type 17 T helper cells negatively correlated with immature dendritic cells (cor = -0.44, p < 0.05) (**Figure 7C**). MRPL53 expression positively correlated with immature dendritic cell infiltration (cor = 0.63, p < 0.01) but inversely with type 17 T helper cells (cor = -0.33, p < 0.05). RPL24 showed positive correlations with central memory CD4 T cells (cor = 0.69, p < 0.001) and negative associations with type 17 T helper cells (cor = -0.59, p < 0.001) (**Figure 7D**). These results highlight disrupted immune microenvironment homeostasis in MDD.

### 3.10 Molecular regulatory network of AICD-related key genes

**Figure 8:**
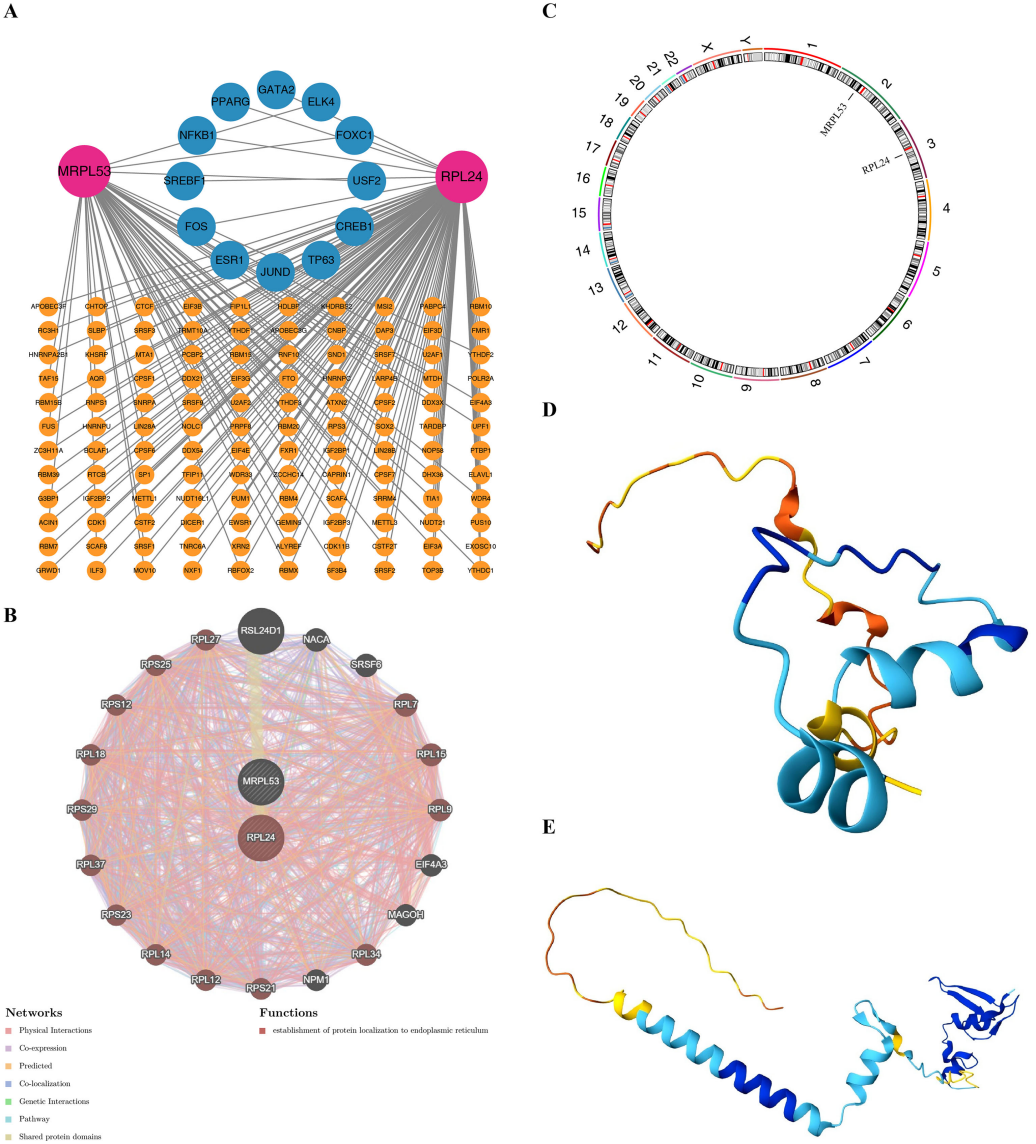
Molecular regulatory network and structural characterization of key genes. **(A)** TF-mRNA-RBP regulatory network for MRPL53 and RPL24. Blue circles: transcription factors; pink circles: key genes; orange hexagons: RNA-binding proteins. **(B)** GeneMANIA functional interaction network showing physical interactions, co-expression, predicted interactions, co-localization, genetic interactions, pathways, and shared protein domains. **(C)** Chromosomal localization of MRPL53 (chromosome 2) and RPL24 (chromosome 3) visualized by Circos plot. **(D, E)** AlphaFold-predicted 3D protein structures of MRPL53 **(D)** and RPL24 **(E)**. Color gradient represents pLDDT confidence score from blue (low) to red (high).

Analysis of the two key genes (MRPL53 and RPL24) identified 120 RBPs through the StarBase v3.0 database and 12 TFs via the JASPAR database. The resulting TF-mRNA-RBP network revealed a complex interplay of transcriptional and post-transcriptional regulators (**Figure 8A**). This network contained 184 pairs of relationships. For example, MRPL53-ACIN1, RPL24-ACIN1, FOXC1-MRPL53, NFKB1-RPL24. This integrative network analysis highlighted the complex regulatory landscape of AICD-related key genes, indicating that transcriptional regulation contributes to the pathogenesis of MDD.

The gene interactions and functional landscape of key genes were further revealed. The key gene and 20 similar genes mainly interact with physical interactions, co-expression, predicted, co-localization, genetic interactions, pathway, shared protein domains **(****Figure 8B**).

### 3.11 Chromosomal localization and structural characterization of key genes

Chromosomal localization mapped MRPL53 to chromosome 2 and RPL24 to chromosome 3 (**Figure 8C**). These loci overlap with regions implicated in neurodevelopmental disorders (35), suggesting functional relevance to MDD. Structural analysis revealed that MRPL53 contains a conserved mitochondrial ribosomal large subunit domain critical for ribosomal assembly and oxidative phosphorylation (**Figure 8D**), while RPL24 displays a ribosomal protein L24e domain essential for translational regulation (**Figure 8E**). Both structures exhibited pLDDT scores exceeding 70, validating structural reliability.

### 3.12 Identification of potential therapeutic agents targeting key genes

**Figure 9:**
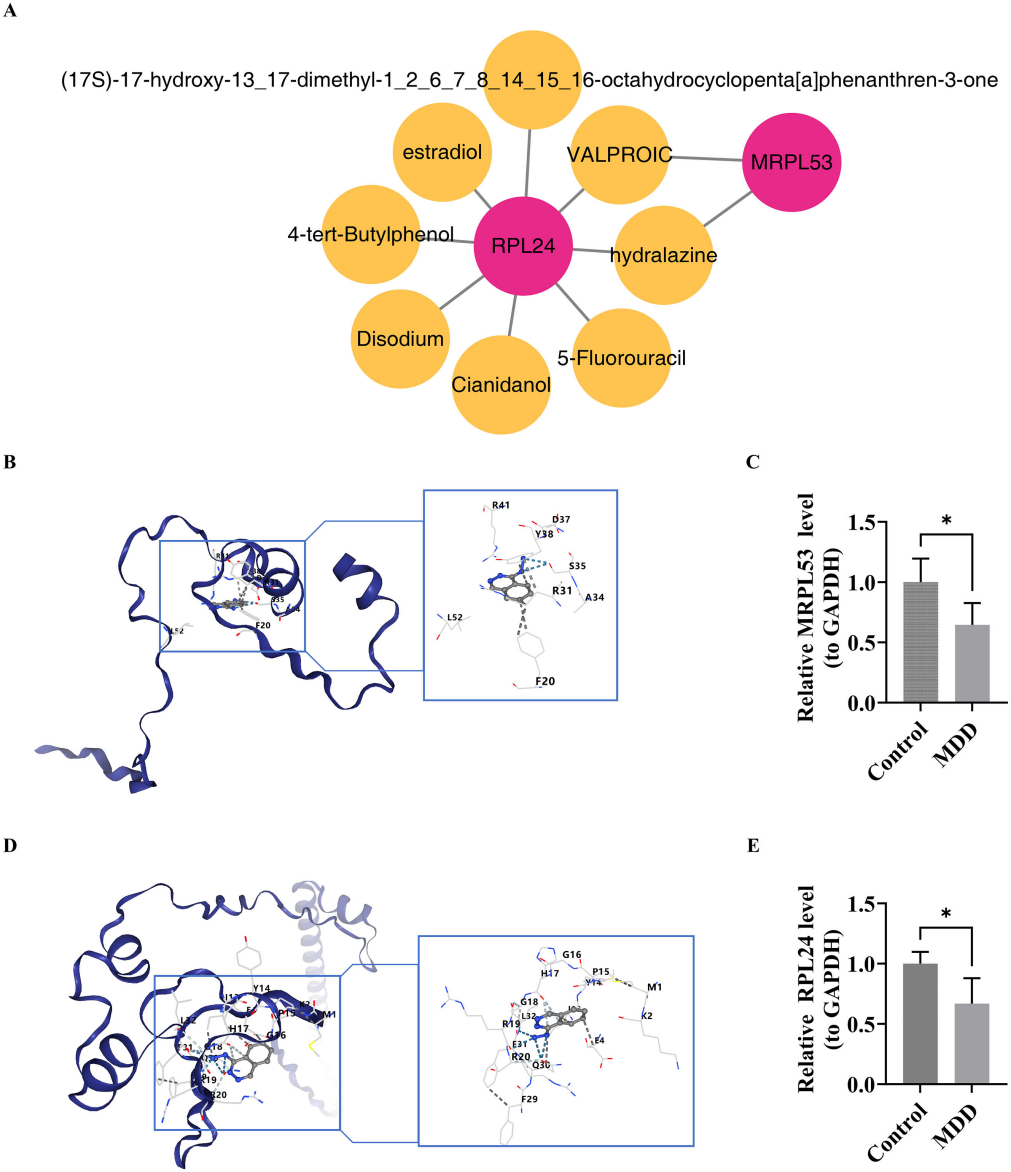
Drug prediction, molecular docking, and experimental validation. **(A)** Drug-gene interaction network showing predicted therapeutic agents for MRPL53 and RPL24. Pink circles: key genes; orange circles: candidate drugs. Hydralazine shows interactions with both genes. **(B, D)** Three-dimensional molecular docking models showing binding interactions between hydralazine and MRPL53 [**(B)**, binding affinity = -5.0 kcal/mol] and RPL24 [**(D)**, binding affinity = -5.7 kcal/mol]. Left panels: overall binding poses; right panels: detailed views showing key residues and hydrogen bonds (dashed lines). **(C, E)** RT-qPCR validation showing significant downregulation of MRPL53 and RPL24 in MDD patients compared to controls (*p < 0.05, t-test).

Identifying potential drugs with therapeutic potential for MDD, and analyzing the interactions of these drugs with key genes MRPL53 and RPL24 (**Figure 9A**). Drug prediction via the DSigDB database revealed two candidate drugs for MRPL53 and eight for RPL24, with hydralazine (a vasodilator used in hypertension management) ranking as the top-scoring agent for both genes (Combined Score: 1st rank). The analysis of molecular docking revealed that hydralazine exhibited a strong affinity for the key genes, with docking scores of -5.0 kcal/mol (MRPL53) and -5.7 kcal/mol (RPL24), indicating that the interactions were stable (**Table 1**, **Figures 9B, D**). These results represent a preliminary in silico hypothesis only and require further functional validation.

**Table 1:** Molecular docking results of candidate drugs with key genes MRPL53 and RPL24.

MOL ID	Molecular name	Gene name	AF ID	Score
3637	hydralazine	MRPL53	AF-B9A051-F1	-5
3637	hydralazine	RPL24	AF-P83731-F1	-5.7

### 3.13 Expression levels of key genes

To validate the bioinformatics predictions, the mRNA expression levels of MRPL53 and RPL24 were quantitatively analyzed in MDD patients (n = 5) and controls (n = 5) using RT-qPCR. Significant differences in gene expression were observed between the two cohorts. Specifically, compared to the control samples, the MDD samples exhibited marked downregulation of MRPL53 and RPL24 expression (p < 0.05) (**Figures 9C, E**). These differential expression patterns aligned with predictions from prior bioinformatics analyses, thereby providing preliminary support for the hypothesis that these genes might contribute to the pathogenesis of MDD.

## 4 Discussion

MDD is a complex neuropsychiatric disorder involving genetics, neurobiology, and immune metabolism (3, 36). AICD, as a key link in energy metabolism imbalance, may influence MDD progression by regulating mitochondrial function and immune response (37). This study identified MRPL53 and RPL24 as AICD-associated transcriptomic markers in MDD through bioinformatics approaches and demonstrated their diagnostic performance. Enrichment analysis suggested these genes affect MDD progression by regulating protein synthesis and energy metabolism. Immune infiltration analysis revealed significant alterations in the immune microenvironment of MDD patients, with key genes showing strong correlations with specific immune cell populations.

MRPL53 (mitochondrial ribosomal protein L53) is a constituent of the mitochondrial ribosome large subunit. Its function is regulated through post-transcriptional modifications and RNA binding mechanisms that control mitochondrial protein synthesis efficiency (38). This protein participates in facial morphogenesis through interaction with MYC transcription factor and acts as a key factor in mitochondrial ribosome assembly (39). MRPL53 regulates macrophage polarization and exhibits tissue-specific expression in inflammatory diseases (40). In this study, MRPL53 was significantly downregulated in MDD patients, likely disrupting mitochondrial protein synthesis and energy metabolism while causing immune system imbalance that intensifies neuroinflammatory responses.

RPL24 (ribosomal protein L24) is a constituent of the ribosome large subunit (60S). Through interactions with 28S rRNA and other ribosomal proteins like RPL5 and RPL11, it participates in ribosome assembly and protein synthesis regulation (41). RPL24 mutations are associated with Diamond-Blackfan anemia, characterized by ribosome dysfunction (42, 43) RPL24 may affect cellular stress responses and immune signaling pathways (44). RPL24 was also significantly downregulated in MDD patients, potentially decreasing ribosome assembly efficiency and affecting immune cell functions.

In conclusion, the downregulation of MRPL53 and RPL24 suggests broader mitochondrial and translational dysregulation, rather than direct involvement in the ATP-P2X7R-NLRP3 AICD axis in MDD patients, providing new insights into MDD molecular mechanisms.

The nomogram model constructed based on the MRPL53 and RPL24 genes in this study demonstrated outstanding discriminatory performance in the training cohort, with an area under the receiver operating characteristic curve (AUC) reaching as high as 0.967, significantly surpassing the minimum effective threshold (AUC > 0.8) for psychiatric biomarkers with clinical application potential recommended by the American Psychiatric Association (APA) working group (45). The discriminatory ability of this model was also significantly higher than that of most current biomarker studies related to depression (MDD)-the typical AUC range of these latter studies was mostly concentrated between 0.63 and 0.85. For instance, Zhang et al. (2023) reported that the AUC value of circulating protein biomarkers in the diagnosis of MDD exceeded 0.85 (46). The AUC values obtained by the longitudinal study of precursory symptoms in North America in the task of predicting mental illness ranged from 0.63 to 0.79 (45). In the independent validation cohort, the nomogram model based on these two genes still maintained good discriminatory efficacy (the AUC values corresponding to the two genes were both > 0.7), confirming the good cross-cohort reproducibility of the model and effectively addressing the common overfitting problem in biomarker research - a problem that often limits the practical application of biomarkers (45). However, the slight decline in performance from the training cohort to the validation cohort also reflects the inherent complexity and challenges in the development of psychiatric biomarkers. These include biological heterogeneity and the significant impact of demographic factors (such as age, gender, etc.) on the accuracy of model discrimination (47). It is notable that recent studies have suggested that stratified analysis by gender can further enhance the discrimination performance of the model: when the data are modeled separately by gender, the AUC value can increase from 0.739 to 0.806-0.807. This discovery has provided us with significant guidance for the optimization direction of our subsequent models.

This study identified an increase in the abundance of memory B cells and immature dendritic cells in the peripheral blood of patients with Major Depressive Disorder (MDD), while a significant decrease in Th17 cells was observed (p < 0.05). This phenomenon is consistent with the findings in animal models that stress-induced excessive activation of B cells leads to aggravated neuroinflammation (48). The immune microenvironment is an important system that maintains the body’s immune homeostasis and responds to pathogen invasion. The alterations in this environment observed in patients with MDD suggest that the immune system plays a substantial role in the pathogenesis of MDD. The association between RPL24 expression and ssGSEA-derived estimates of central memory CD4 T cells is consistent with the hypothesis that immunological memory-related transcriptional profiles may be altered in MDD patients. However, as these estimates are derived from bulk peripheral blood transcriptomes using correlation-based deconvolution, causal relationships cannot be inferred. The positive correlation between MRPL53 expression and immature dendritic cell estimates (cor = 0.63) is consistent with a potential link between mitochondrial gene expression and immune profiles observed in previous studies of inflammatory conditions (49, 50). Specifically, MRPL53 expression levels are correlated with the abundance and distribution of immature dendritic cells, aligning with reported associations between mitochondrial gene expression and immune cell characteristics in inflammatory contexts. Changes in its expression level are associated with the maturation state of dendritic cells. The correlation between RPL24 and central memory CD4 T cells (cor = 0.69) is consistent with a potential link between immune memory and MDD pathogenesis (51). These correlational findings from bulk transcriptome deconvolution should be interpreted with caution and do not imply causal relationships.

In this study, molecular docking demonstrated that hydralazine showed moderate binding affinity for MRPL53 (−5.0 kcal/mol) and RPL24 (−5.7 kcal/mol). These results are only preliminary in silico findings without any functional validation. Hydralazine, as a classic antihypertensive drug, has been reported to exhibit DNA methyltransferase inhibitory activity in previous studies. It can exert demethylation effects by reducing the expression of DNMT1 and DNMT3a (52). This dual-targeting mechanism (ribosomal pathway and mitochondrial pathway) may enable hydralazine to exert a synergistic effect in the treatment of MDD. Molecular docking studies have shown that hydralazine forms stable interactions with the Lys162 and Arg240 residues at the active site of DNA methyltransferase, supporting the possibility that it directly inhibits enzyme activity (52). This epigenetic regulatory mechanism holds significant innovative value for the treatment of MDD, given that epigenetic modifications are pivotal in the regulation of gene expression. Hydralazine can restore the normal expression of silenced genes through demethylation (53). Clinical trial evidence indicates that hydralazine combined with epigenetic therapy shows promising clinical benefits in various diseases, further supporting its therapeutic potential in MDD (54, 55). However, it should be noted that hydralazine may cause neuro-psychiatric side effects such as depression or anxiety in some cases (56), which suggests that in clinical applications, the dose-effect relationship and individualized treatment plans need to be carefully evaluated. Future studies should further verify the neuroprotective mechanism of hydralazine on MDD through animal experiments.

In conclusion, this study identifies MRPL53 and RPL24 as candidate genes associated with AICD-related transcriptional profiles in MDD and revealed hydralazine’s antidepressant potential through a dual-targeting mechanism, providing a transcriptomic basis for MDD diagnosis, pending mechanistic validation and drug repositioning. This study has certain limitations, including relatively small sample size, absence of brain tissue-specific validation, and potential selection bias from retrospective data analysis. The molecular docking results require further validation through *in vitro* enzyme activity assays and cell function experiments. In addition, there are differences in the sample types (whole blood vs. peripheral blood mononuclear cells) between the training cohort and the validation cohort. Whole blood contains cell components such as granulocytes and red blood cells that do not exist in PBMC. The transcriptomes and immune characteristics of the two are different. This heterogeneity has significant confounding effects on immune infiltration analysis and may lead to a decline in the cross-cohort diagnostic efficacy of the model. Therefore, conclusions on cross-cohort reproducibility need to be interpreted with caution. Subsequent research can prioritize conducting gene knockdown experiments to verify the direct role of MRPL53 and RPL24 in neuronal survival, constructing animal models of MDD to evaluate the antidepressant efficacy and optimal dose of hydralazine, and combining single-cell sequencing to analyze the expression patterns of key genes in different brain regions and cell types. At the same time, matching sample types (uniformly using whole blood or PBMC) should be adopted to verify the results of this study, providing a more reliable basis for clinical translation.

## 5 Conclusion

This study identifies MRPL53 and RPL24 as candidate AICD-associated transcriptomic markers in MDD through integrated transcriptomic analysis, WGCNA, and machine learning. They demonstrated excellent diagnostic performance in both training and validation cohorts (AUC > 0.7), and the diagnostic nomogram constructed based on them showed high accuracy (AUC = 0.967). Functional enrichment analysis indicated these genes may contribute to MDD pathogenesis by regulating pathways such as ribosome biogenesis, neuroactive ligand-receptor interaction, and oxidative phosphorylation. Immune infiltration analysis revealed significant imbalance in the immune microenvironment of MDD patients, with strong correlations between key gene expression and specific immune cell populations. Molecular docking predicted hydralazine as an in silico ligand for MRPL53 and RPL24. These computational findings provide a hypothesis-generating basis for future experimental validation, but do not constitute evidence for drug repurposing. RT-qPCR validated the differential expression of key genes in clinical samples. In conclusion, this study highlights potential mitochondrial and ribosomal involvement in MDD, without claiming direct AICD pathway membership and provides supportive evidence for exploring potential diagnostic biomarkers and preliminary therapeutic clues for MDD.

## Data Availability

All gene expression datasets used in this study are publicly available in the Gene Expression Omnibus (GEO) repository at https://www.ncbi.nlm.nih.gov/geo/. The training dataset (GSE52790, GPL17976 platform) and validation dataset (GSE38206, GPL13607 platform) can be freely accessed and downloaded. The 37 AICD-related genes were sourced from 14. Supplementary data are provided with this article. Any additional information required to reanalyze the data reported in this paper is available from the corresponding author upon request.
