# Supplementary figures and images for: Identification of AICD-associated transcriptomic markers in major depressive disorder
Source: Front Psychiatry. 2026 Jul 3;17:1782515. doi: 10.3389/fpsyt.2026.1782515 (PMC13376302; doi:10.3389/fpsyt.2026.1782515)

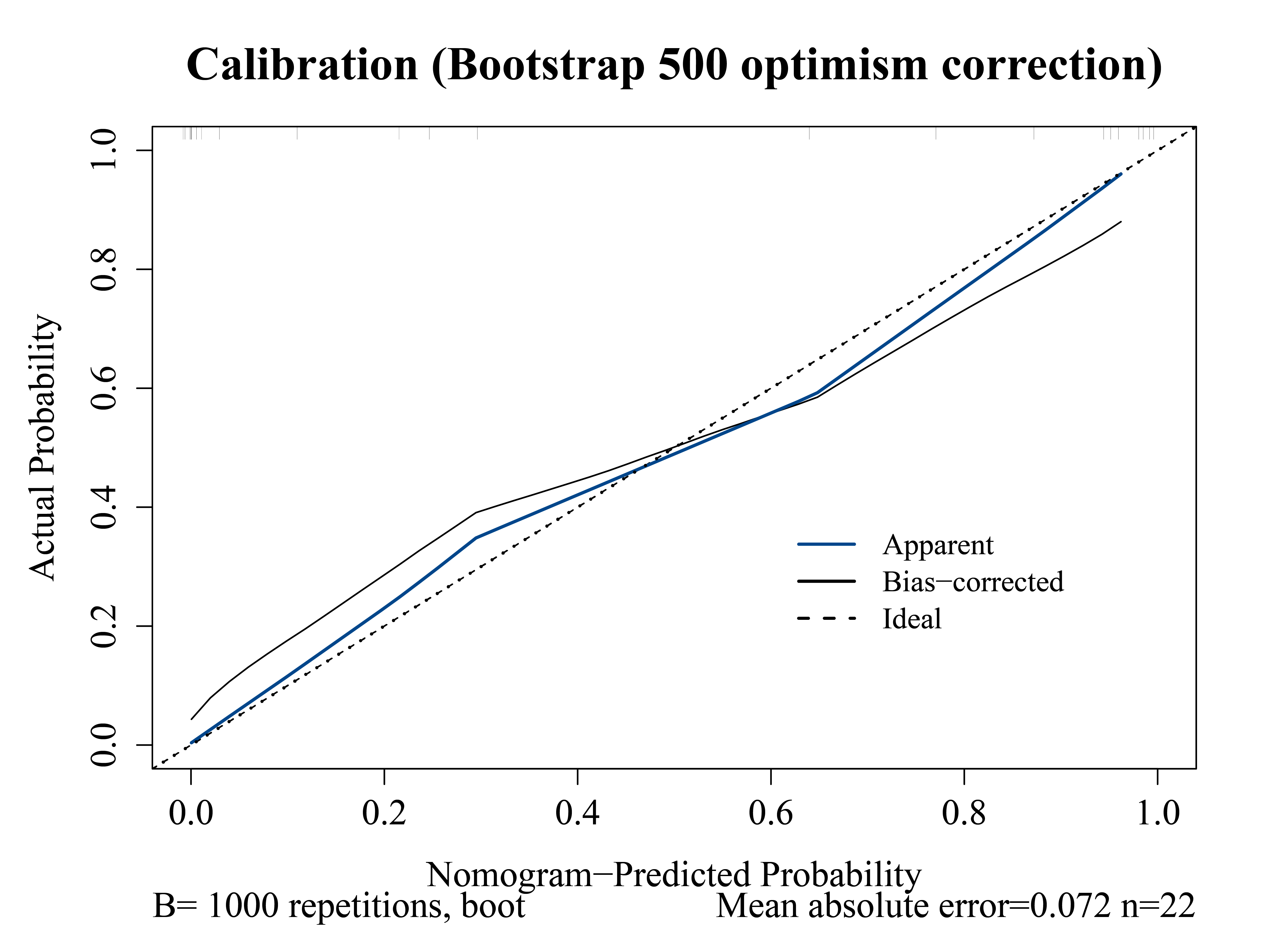

Supplement: Supplementary file 1 [file Image1.tif]
