# Supplementary material for: Identification of AICD-associated transcriptomic markers in major depressive disorder
Source: Front Psychiatry. 2026 Jul 3;17:1782515. doi: 10.3389/fpsyt.2026.1782515 (PMC13376302; doi:10.3389/fpsyt.2026.1782515)
Supplement: Supplementary file 5 [file Table3.docx]

Supplementary Material

# Supplementary Tables

**Supplementary Table 1. The list of AICD-related genes**

**Supplementary Table 2. Primer sequences used for RT-qPCR validation**

# Supplementary **Figure**

**Supplementary Figure 1. Calibration curve.** The x-axis represents the nomogram-predicted probability of MDD, and the y-axis represents the observed probability of MDD. The blue line indicates the relationship between predicted and observed probabilities. The black solid line represents the bias-corrected calibration curve, and the black dashed line represents the ideal calibration curve. Bootstrap with 1000 repetitions was used for model validation. Mean absolute error (MAE) measures the average difference between predicted and observed values; lower MAE indicates higher predictive accuracy.
